# Natural variation in root exudate composition in the genetically structured *Arabidopsis thaliana* in the Iberian Peninsula

**DOI:** 10.1111/nph.20314

**Published:** 2024-12-10

**Authors:** Harihar Jaishree Subrahmaniam, F. Xavier Picó, Thomas Bataillon, Camilla Lind Salomonsen, Marianne Glasius, Bodil K. Ehlers

**Affiliations:** ^1^ Department of Ecoscience Aarhus University Aarhus C 8000 Denmark; ^2^ Institut für Pflanzenwissenschaften und Mikrobiologie Universität Hamburg Hamburg 22609 Germany; ^3^ Departamento de Ecología y Evolución, Estación Biológica de Doñana Consejo Superior de Investigaciones Científicas Sevilla 41092 Spain; ^4^ Department of Molecular Biology and Genetics, Bioinformatics Research Centre Aarhus University Aarhus C 8000 Denmark; ^5^ Department of Chemistry Aarhus University Aarhus C 8000 Denmark

**Keywords:** *Arabidopsis thaliana*, drivers of natural variation, genetic clusters, genome‐wide association analysis, root exudates, terpenoids

## Abstract

- Plant root exudates are involved in nutrient acquisition, microbial partnerships, and inter‐organism signaling. Yet, little is known about the genetic and environmental drivers of root exudate variation at large geographical scales, which may help understand the evolutionary trajectories of plants in heterogeneous environments.
- We quantified natural variation in the chemical composition of *Arabidopsis thaliana* root exudates in 105 Iberian accessions. We identified up to 373 putative compounds using ultra‐high‐performance liquid chromatography coupled with mass spectrometry. We estimated the broad‐sense heritability of compounds and conducted a genome‐wide association (GWA) study. We associated variation in root exudates to variation in geographic, environmental, life history, and genetic attributes of Iberian accessions.
- Only 25 of 373 compounds exhibited broad‐sense heritability values significantly different from zero. GWA analysis identified polymorphisms associated with 12 root exudate compounds and 26 known genes involved in metabolism, defense, signaling, and nutrient transport. The genetic structure influenced root exudate composition involving terpenoids. We detected five terpenoids related to plant defense significantly varying in mean abundances in two genetic clusters.
- Our study provides first insights into the extent of root exudate natural variation at a regional scale depicting a diversified evolutionary trajectory among *A. thaliana* genetic clusters chiefly mediated by terpenoid composition.

## Introduction

Plant root exudates encompass a vast array of primary (e.g. carbohydrates, amino acids, and organic acids) and secondary metabolites (e.g. flavonoids, terpenoids, and alkaloids) that shape the physical, chemical, and biological properties of the soil (Oburger & Jones, 2018). They also facilitate nutrient cycling and mediate biotic interactions in the rhizosphere, thereby fostering a healthy soil ecosystem (Badri & Vivanco, 2009; Rasmann & Hiltpold, 2022). Despite the ecological relevance of root exudates, several factors, such as stress and developmental status, influence their chemical composition and challenge their quantification. For instance, stress by elevated phosphorus increases pthalic acid in *Cyperus alternifolius* (Duan *et al*., 2020), hydric stress induces various organic acids in *Zea mays* (Song *et al*., 2012), and pathogen infection in *Arabidopsis thaliana* stimulates long‐chain fatty acids and amino acids that recruit protective *Pseudomonas* species (Wen *et al*., 2021). Furthermore, development also affects exudate profiles in *A. thaliana* with sugar alcohols decreasing and amino acids increasing over time in early developmental stages (Chaparro *et al*., 2013), whereas young fir trees exudate more carbohydrates and quercetin than older trees, which secrete more lipids and salicylic acids, shifting from nutrient acquisition to defense over‐development (Chen *et al*., 2023). Given the influence of root exudates on plant–environment interactions and adaptive strategies (Novoplansky, 2019; Williams & de Vries, 2020; Subrahmaniam *et al*., 2023), unraveling the chemistry of root exudates may help decipher the complexity of plant metabolism but also the ecology of plant communities (Mommer *et al*., 2016; van Dam & Bouwmeester, 2017; McLaughlin *et al*., 2023).

However, our knowledge of natural variation in root exudate composition is rather scarce (Vives‐Peris *et al*., 2020; Escolà Casas & Matamoros, 2021; Wang *et al*., 2021). This is a problem because understanding natural variation in plant traits is of paramount importance in different disciplines, as natural variation reflects long‐term evolutionary dynamics, can reveal environmental factors driving this variation, and facilitates the exploration of the genetic basis of trait differences (Mitchell‐Olds & Schmitt, 2006; Alonso‐Blanco *et al*., 2009). One reason for the scarcity of studies on natural variation in plant root exudates has to do with the technical challenges for capturing and analyzing the complex chemical data from root exudates (van Dam & Bouwmeester, 2017; Oburger & Jones, 2018). In fact, few studies have described natural variation in chemical composition of root exudates in various plant species (Micallef *et al*., 2009; Biedrzycki *et al*., 2010; Badri *et al*., 2012; Houshyani *et al*., 2012; Chaparro *et al*., 2013; Fang *et al*., 2013; Strehmel *et al*., 2014; Mönchgesang *et al*., 2016; Kawasaki *et al*., 2018; Liu *et al*., 2020), all of them using low sample sizes (< 20 accessions in all cases). Interestingly, recent developments in mass spectrometry and nuclear magnetic resonance spectroscopy now enable the characterization and quantification of specific chemical compounds present in root exudates from a large number of samples (Pantigoso *et al*., 2021; Wang *et al*., 2022).

In this study, we took advantage of such recent technical advances, combined with the availability of dense collections of natural accessions exceptionally well characterized at the ecological, phenotypic, and genomic levels of the annual plant *Arabidopsis thaliana*, to conduct the first regional‐scale assessment of natural variation in root exudate composition in plants with a large sample size. We analyzed root exudates from 105 distinct natural accessions of *A. thaliana* from the Iberian Peninsula. The Iberian collection of *A. thaliana* is geographically structured into four differentiated genetic clusters (Picó *et al*., 2008; Castilla *et al*., 2020), reflecting the complexity of the demographic and evolutionary history of this species across the region. Besides, the Iberian collection contains a remarkably high genetic and phenotypic diversity, including adaptive variation in life‐history traits (Picó *et al*., 2008; Méndez‐Vigo *et al*., 2011; Marcer *et al*., 2018; Tabas‐Madrid *et al*., 2018; Castilla *et al*., 2020) and the highest genomic diversity from the species' native Eurasian range (The 1001 Genomes Consortium, 2016).

Here, we quantified the extent of chemical variation in root exudates across Iberian *A. thaliana* accessions by combining ultra‐high‐performance liquid chromatography with quadrupole time‐of‐flight mass spectrometry (Subrahmaniam *et al*., 2023). We estimated the broad‐sense heritability values of root exudates, thereby assessing their degree of genetic determination. Furthermore, by conducting genome‐wide association (GWA) analyses, we also provided insights into the genetic basis of regional‐scale variation in root exudates. Finally, we examined the eco‐evolutionary forces putatively driving natural variation in root exudates by correlating chemical variation among accessions with their geographic, environmental, life history, and genetic patterns of variation across the Iberian Peninsula.

## Materials and Methods

### Source accessions

The accessions included in this study are part of the Iberian collection of *Arabidopsis thaliana* (L.) Heynh., encompassing hundreds of populations from the southwestern Mediterranean Basin (Spain, Portugal, and Morocco; Picó *et al*., 2008; Brennan *et al*., 2014; Castilla *et al*., 2020). We sampled populations in annual field campaigns in spring from the early 2000s to late 2010s. In each field campaign, seeds from several individuals per population (*c*. 6–7) were collected and multiplied using the single seed descent method in glasshouse conditions (Centro Nacional de Biotecnología, Madrid, Spain) to increase seed number and quality while minimizing environmental and maternal effects. During these multiplication experiments, and when possible, we selected one maternal line per population based on average values per population for adaptively important life‐history traits (e.g. flowering time). These maternal lines became accessions used for characterizing phenotypic traits and obtaining whole‐genome sequences in subsequent studies (Tabas‐Madrid *et al*., 2018). We kept multiplied seeds in cellophane bags under dry conditions, at room temperature, and in darkness for long‐term storage. To avoid aging effects, seeds used in this study came from an additional multiplication experiment conducted in the late 2010s.

Previous genetic structure analysis on the Iberian *A. thaliana* collection revealed the existence of four genetic clusters: NW‐C1, NE‐C2, Relict‐C3, and SW‐C4 (cluster names as in Castilla *et al*., 2020) (Supporting Information Dataset S1). The three nonrelict clusters are geographically structured as indicated by cardinal directions in their names, while the relict cluster is scattered mostly across the southern half of the Iberian Peninsula (Marcer *et al*., 2016). Evidence indicates that the relict cluster, which has several unique phenotypic and molecular features, probably endured dramatic environmental changes over millennia, representing part of the species' early history (Durvasula *et al*., 2017; Toledo *et al*., 2020). The Iberian *A. thaliana* collection is also well characterized ecologically (Marcer *et al*., 2016; Vidigal *et al*., 2016; Tabas‐Madrid *et al*., 2018; Castilla *et al*., 2020) (Dataset S1), which is important for this study. In short, geographic coordinates and altitude of all accessions were recorded during field campaigns with a GPS (Garmin International Inc., Olathe, KS, USA; positional error = 4 m). Climatic data associated with accession coordinates were extracted from worldclim v.2 (Fick & Hijmans, 2017) and the Digital Climatic Atlas from the Iberian Peninsula (http://opengis.uab.es/wms/iberia/en_index.htm). Vegetation data came from the CORINE Land Cover 2000 (https://land.copernicus.eu/pan‐european/corine‐land‐cover) as the percentage of vegetation types within a 500 m radius around the GPS coordinates of each accession. Finally, topsoil pH came from The Soil Geographical Database from eurasia v.4 (https://esdac.jrc.ec.europa.eu/tags/soil‐geographical‐database‐eurasia).

### Plant growth and root exudate sample collection

We initially selected 131 natural *A. thaliana* accessions from four genetic clusters, located in areas with < 50% urbanized land (Marcer *et al*., 2016). Using a majority rule for genetic cluster memberships, the sample set included 91 accessions from NW‐C1, 17 from NE‐C2, 14 from Relict‐C3, and 9 from SW‐C4. This distribution of accessions reflects the natural abundance of *A. thaliana* in areas with low human influence for each genetic cluster. Clusters with a broad distribution, such as NW‐C1, span diverse environments, whereas those with narrower distributions, such as SW‐C4, are more specific to environmental conditions from smaller regions.

In July–September 2022, seeds from each accession were grown in five replicates at the Department of Molecular Biology and Genetics (Aarhus University, Aarhus, Denmark) using a protocol for handling large samples (Subrahmaniam *et al*., 2023). Briefly, all plants were grown *in vitro* at 21°C with a 16 h : 08 h, light : dark cycle. Each Petri plate (total 153 plates) containing Murashige and Skoog medium with agar housed four to five accessions and one control. Plants were grown on autoclaved polypropylene mesh for facilitating easy removal. All plates were rotated periodically to minimize position effects. Col‐0 was also used as a phytometer to gauge micro‐environmental variation. All accessions were sampled 6 wk after germination, during a specified period in the day (between 08 : 00 h and 11 : 00 h) to minimize diurnal variations. For exudate collection, each plant was removed using the meshes, roots were cleaned with a brush, and then transferred to 400 μl of MilliQ (0.5 μS cm^−1^, Millipore, Merck KGaA, Darmstadt, Germany) water for 5 min to capture any erroneous exudates. Individual plants were then moved to 400 μl MilliQ water in 20 ml cylindrical glass vials, with roots submerged and the aboveground part floating with the help of the mesh. Vials were sealed to ensure sterility and trays containing vials were covered with aluminum foil at the base to simulate dark conditions. Trays were then placed in a growth chamber at 21°C for 2 h. Afterwards, plants were removed from the vials and samples were immediately frozen in liquid nitrogen and stored at −18°C until further processing. Negative controls were prepared by treating empty vials following the same procedure.

### Root exudate chemical analysis

**Fig. 1:**
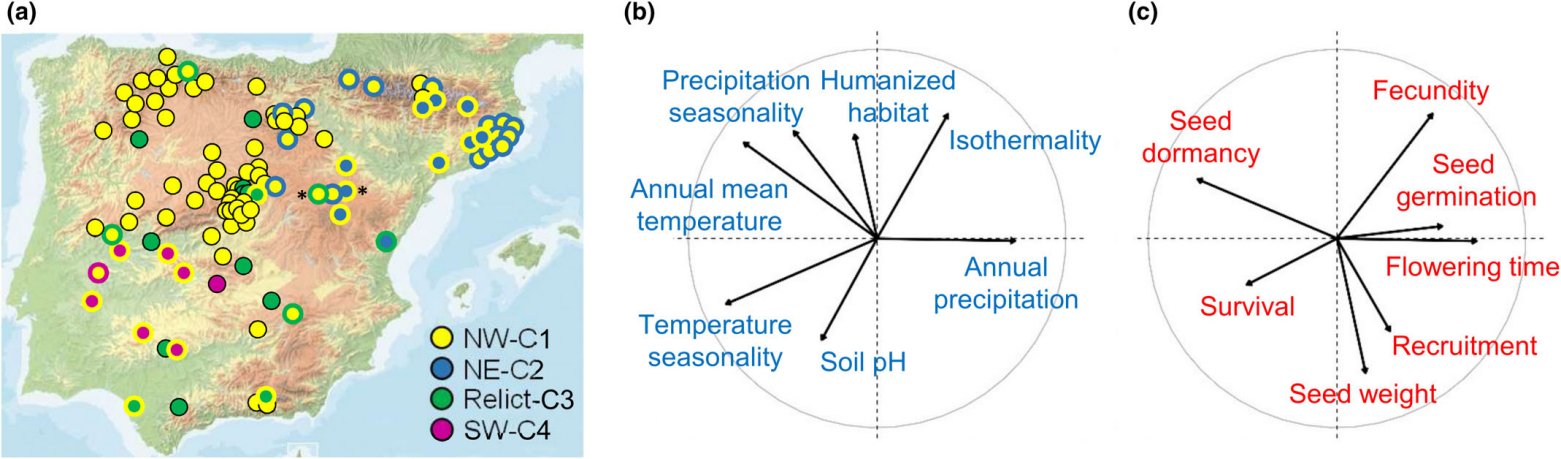
Geographic, genetic, environmental, and life‐history diversity of Iberian *Arabidopsis thaliana* accessions used to estimate root exudate composition (see Supporting Information data in Dataset S1). (a) Geographic distribution of 105 accessions across the Iberian Peninsula. Accessions are color‐coded based on the assigned genetic cluster as follows. Dots with a unique color indicate accessions with proportional memberships higher than 0.5 in a single cluster and lower than 0.25 in the other three clusters. Dots with two colors indicate accessions with a predominant membership in a single cluster (inner color) and higher than 0.25 in another cluster (outer color). Only two accessions exhibited proportional memberships higher than 0.25 in three clusters (indicated by asterisks). In these two cases, we indicate the proportional memberships of the two main clusters following the same rationale as before. (b, c) Principal component analysis (PCA) plots of environmental variables (b) and life‐history traits (c) characterizing all 105 accessions. Plots indicate the first two PCA axes and arrow length is proportional to the contribution of each variable/trait.

Overall, we retained 378 plant samples, comprising three to four replicates for 105 out of the 131 accessions, due to the loss of plants during growth and/or the hydroponic root exudate sampling. Based on majoritarian genetic cluster membership, the final set of 105 accessions encompassed 75 accessions from NW‐C1 (mean cluster membership proportion ± SE = 0.70 ± 0.01; range = 0.37–0.90), 10 from cluster NE‐C2 (mean ± SE = 0.55 ± 0.03; range = 0.40–0.70), 13 from cluster Relict‐C3 (mean ± SE = 0.77 ± 0.02; range = 0.64–0‐88), and 7 from cluster SW‐C4 (mean ± SE = 0.59 ± 0.03; range = 0.50–0.68). Some accessions exhibited mixed levels of membership consistent with some degree of admixture among genetic clusters (Fig. 1a), which provides a snapshot of the demographic and evolutionary complexity of *A. thaliana* across the region. It must be noted that the placement of accessions among clusters was not a biasing factor in this study design because we instead used genetic cluster proportional memberships in our analyses as a covariate (to be described later).

In September–December 2022, samples were subjected to untargeted metabolic profiling in negative and positive ionization modes using ultra‐high‐performance liquid chromatography coupled with quadrupole time‐of‐flight mass spectrometry (UHPLC‐QTOF‐MS) at the Department of Chemistry of Aarhus University (Aarhus, Denmark). The UHPLC was an Ultimate 3000 (Thermo Scientific Dionex, Sunnyvale, CA, USA) and the QTOF‐MS was a Bruker Compact (Bruker, Billerica, MA, USA). Instrument stability was assessed using a standard solution of six authentic standards (cinnamic acid, naringenin, L‐tryptophan, quercetin, salicylic acid, and p‐coumaric acid) injected throughout the run (Broadhurst *et al*., 2018). Additionally, an internal standard of ketopinic acid (10 μg ml^−1^ in MilliQ water) was prepared for spiking all samples. Quality control samples were injected every 30 samples and biological control blanks every 20 samples. The *m/z* error averaged < 3 ppm, retention time error was < 1%, and peak area error was < 10% in negative mode. In positive mode, only L‐tryptophan was adequately ionized, with an m/z error < 2 ppm, retention time error < 1%, and peak area error < 10%, indicating no significant instrumental drift throughout both runs. However, quercetin, known to be photosensitive, exhibited greater variability in signal stability over time. Despite these fluctuations, the signals remained within the predefined warning and action thresholds so that the variability was not due to analytical instability.

### Raw data processing

We processed the raw LC‐MS data using MZmine 3 (Schmid *et al*., 2023) and SIRIUS 5 (Dührkop *et al*., 2021). MZmine 3 performs preprocessing, peak detection, alignment, quantification, and statistical analysis for untargeted metabolic profiling (Ludwig *et al*., 2018). The features were analyzed with SIRIUS 5, which uses isotopic pattern and fragmentation trees for structure elucidation and chemical annotation. Specifically, SIRIUS 5 uses CSI:FingerID and CANOPUS to predict molecular fingerprints and compound classes (Ludwig *et al*., 2020; Dührkop *et al*., 2021). CANOPUS integrates ClassyFire and Natural Product Classifier (NPC) to assign compound classes with a flexible three‐tiered system (pathway, superclass, and class), allowing compounds to fit into multiple categories (Kim *et al*., 2021) while providing a Tanimoto similarity for structural matching (Ludwig *et al*., 2020). In evaluating the putative chemical compound prediction, a combination of SIRIUS chemical classes and Tanimoto scores has been endorsed (Zulfiqar *et al*., 2023).

In total, we detected 1142 features in negative ionization mode and 126 in positive mode across 105 Iberian *A. thaliana* accessions. Of these, 992 and 108 were identified as putative chemical compounds down to the NPC pathway level in negative and positive ionization modes, respectively, while up to 150 (*c*. 13%) and 18 (*c*. 14%) unidentified features were excluded in negative and positive modes, respectively. No features overlapped between negative and positive modes due to the high noise threshold imposed and the lower number of features obtained in positive mode.

### Data filtering for analysis

We converted absolute peak area data of features from MZmine 3 into relative data by dividing absolute peak area of a feature by the total peak area within the respective sample to estimate the contribution of each feature to the entire exudate composition of each sample. We excluded features present in only one replicate of any accession, eliminating 345 (*c*. 30%) and 24 (*c*. 19%) features in the negative and positive modes, respectively. Additionally, to avoid bias, features that were present in either < 10% or > 90% of the accessions – 470 (*c*. 41%) in negative and 56 (*c*. 44%) features in positive mode – were also omitted due to their rarity or prevalence, respectively. This filtering process resulted in 327 features in negative mode and 46 in positive mode for further analysis. Of these final 373 features, 358 (*c*. 95%) were successfully annotated at the compound class level using CANOPUS, with 125 (*c*. 33%) having a Tanimoto score > 50%, displaying structural similarity to known compounds (Ludwig *et al*., 2012) (Dataset S2). For clarity in subsequent sections, we refer to these 373 features as putative compounds as their identity has not been confirmed using authentic standards.

### Broad‐sense heritability values

For each of the 373 compounds, we estimated the broad‐sense heritability (*H* ^2^) as *V*_B_/(*V*_B_ *+ V*_E_), where *V*_B_ is the among‐accession variance and *V*_E_ is the environmental variance (among individual replicates within each accession) for the relative compound abundance (Dataset S2). To estimate *V*_B_ and *V*_E_, we employed a linear mixed model framework where compound variation (response variable) was modeled as a function of accession identity (random effect). Restricted maximum likelihood (REML) estimation was used to obtain variance component estimates using the *lmer* function from the *lme4* R package (Bates *et al*., 2015). By fitting the model, we obtained estimates of the variance among accessions (*V*_B_) and the total variance (*V*_B_ *+ V*_E_). To quantify uncertainty in *H* ^2^ estimates, we used a bootstrap approach in which resampling was conducted at the accession level. For each bootstrap dataset, a linear mixed‐effects model was fitted and *H* ^2^ recalculated. We then used the bootstrap distribution of *H* ^2^ estimates to obtain 95% confidence intervals (using quantiles of the bootstrap distribution). We added a small constant value (1 × 10^−10^) to the relative peak area values for each feature individual measurements before analysis. This adjustment ensured that no values became exactly zero, as it could lead to computational challenges.

### Distance matrices and analyses

We estimated the Bray–Curtis chemical distance among the 105 accessions using the relative peak area of the 373 compounds with the vegan R package (https://CRAN.R‐project.org/package=vegan). Chemical distance among accessions was estimated for all compounds and for each of the seven NPC pathway levels. NPC levels included 59 alkaloids, 108 amino acids and peptides, 88 carbohydrates, 46 fatty acids, 43 shikimates and phenylpropanoids, 9 terpenoids, and 5 polyketides. When analyzing NPC pathways, we excluded 15 compounds because they could not be assigned to any NPC pathway. We additionally computed the chemical distance among accessions based on the first three principal coordinate analysis (PCoA) axes of their variation using the stats R package (R Core Team, 2021).

We estimated the Euclidian geographic distance among accessions using GPS coordinates of each accession (Dataset S1) with passage v.2 (Rosenberg & Anderson, 2011). In the case of genetic distance among accessions, we used whole‐genome sequences previously obtained from 174 Iberian *A. thaliana* accessions including 2.8 million of single‐nucleotide polymorphisms (SNPs) (Tabas‐Madrid *et al*., 2018). Genetic distance was calculated based on the proportions of allele differences between pairs of accessions using all nonsingleton SNPs with tassel v.5.2 (Bradbury *et al*., 2007). Additionally, for each genetic cluster, we computed a genetic cluster distance among accessions based on proportional memberships (estimated in Tabas‐Madrid *et al*., 2018; Dataset S1), resulting in four additional genetic distance matrices. This allowed us to test whether genetic cluster membership affected root exudate composition for all compounds and for compounds classified in each of the seven NPC pathway levels.

We assessed life‐history distance among accessions using quantitative data for life‐history traits obtained from previous studies (Manzano‐Piedras *et al*., 2014; Vidigal *et al*., 2016; Marcer *et al*., 2018) that had pairwise correlation coefficients lower than 0.7. These traits included seed weight, seed dormancy (as quantified by days of seed dry storage required to reach 50% of germination; DSDS50), seed germination in growth chamber conditions at 22°C, and life‐history traits estimated in a common garden experiment, such as recruitment, survival, flowering time and fecundity (see experimental details in Manzano‐Piedras *et al*., 2014; Dataset S1). Combining all phenotypic traits – that directly or indirectly affect fitness in *A. thaliana* – allowed us to relate the phenotypic adaptive attributes of each accession to its environmental conditions of origin. Thus, we first reduced the phenotypic space by conducting a principal component analysis (PCA) on life‐history traits and then computed the Euclidian life‐history distance based on the first three PCA axes (accounting for 65.3% of the variation) with PASSaGE (Fig. 1b).

Finally, we estimated the Euclidian environmental distance among accessions using the environmental datasets available with a similar procedure as for life‐history distance matrix. In short, we first selected environmental variables with pairwise correlation coefficients lower than 0.7 and with a clear biological interpretation. Thus, we eventually considered annual mean temperature, isothermality, temperature seasonality, annual precipitation, precipitation seasonality, the proportion of humanized habitat around each accession, and topsoil pH (Dataset S1). We then estimated the Euclidian environmental distance based on the first three PCA axes (accounting for 71.9% of the environmental variation) with PASSaGE (Fig. 1c).

We explored the drivers of variation in chemical compounds among 105 *A. thaliana* populations by correlating pairwise chemical distance matrices with geographic, environmental, life history, and genetic distance matrices. We used standardized data (by subtracting the mean and scaling by the variance) in all analyses. Analyses were conducted with the whole dataset (373 compounds), the set of compounds with *H* ^*2*^ values significantly different from zero, and for each set of compounds falling into each NPC pathway levels. We conducted Mantel tests with a Bonferroni correction for multiple testing with *vegan*.

### Genome‐wide association analysis

We conducted GWA analyses to identify genomic regions underlying variation in chemical composition of root exudates in the Iberian *A. thaliana* collection. To this end, we conducted GWA analyses using the average relative peak abundances of chemical compounds for each accession as traits with a set of 1.5 million nonsingleton SNPs previously developed for the Iberian *A. thaliana* collection that were functionally annotated (Tabas‐Madrid *et al*., 2018; Arteaga *et al*., 2021). The SNP dataset was filtered to retain only those SNPs possessing a minor allele frequency (MAF) of at least five accessions in the 105 accessions (MAF ≥ 5%). Heterozygous calls in these SNPs were rescored to the major frequency allele and outliers from frequency distributions of traits were eliminated (0.6% of outliers in the final dataset), as they both reduce GWA statistical power (Atwell *et al*., 2010).

To improve the biological interpretation of the results, particularly when known genes were identified, we streamlined the initial list of 373 putative compounds to only those that received a probability score > 0.7 in the NPC pathway, superclass, and class categories. This refinement resulted in 109 putative chemical compounds being included in the GWA analyses from the families of NPC pathway classes (alkaloids: 6; amino acids: 36; carbohydrates: 23; fatty acids: 19; shikimates and phenylpropanoids: 22; and terpenoids: 2; and polyketides: 1). GWA analysis was also conducted using the subset of compounds with *H* ^*2*^ values significantly different from zero.

For the GWA analyses, we employed a mixed linear model (MLM) implemented in TASSEL. To adjust for population structure, we included a genetic kinship matrix, and estimated from the proportion of shared alleles (Atwell *et al*., 2010), as a covariate. SNPs were genotyped as binary variables (0 or 1) and their associations with the root exudate data were assessed using a linear regression model. We applied a stringent significance threshold of –log_10_(*P*) = 7.5 to detect the most significant SNP associations. We used a previous Gene Ontology (GO) annotation enrichment on the Iberian collection (Tabas‐Madrid *et al*., 2018) to identify genes detected by the GWA analyses based on the positions of significant SNPs, considering the two nearest flanking genes in the case of SNPs located in intergenic regions.

## Results

### Chemical variation of *A. thaliana* root exudates in the Iberian collection

**Fig. 2:**
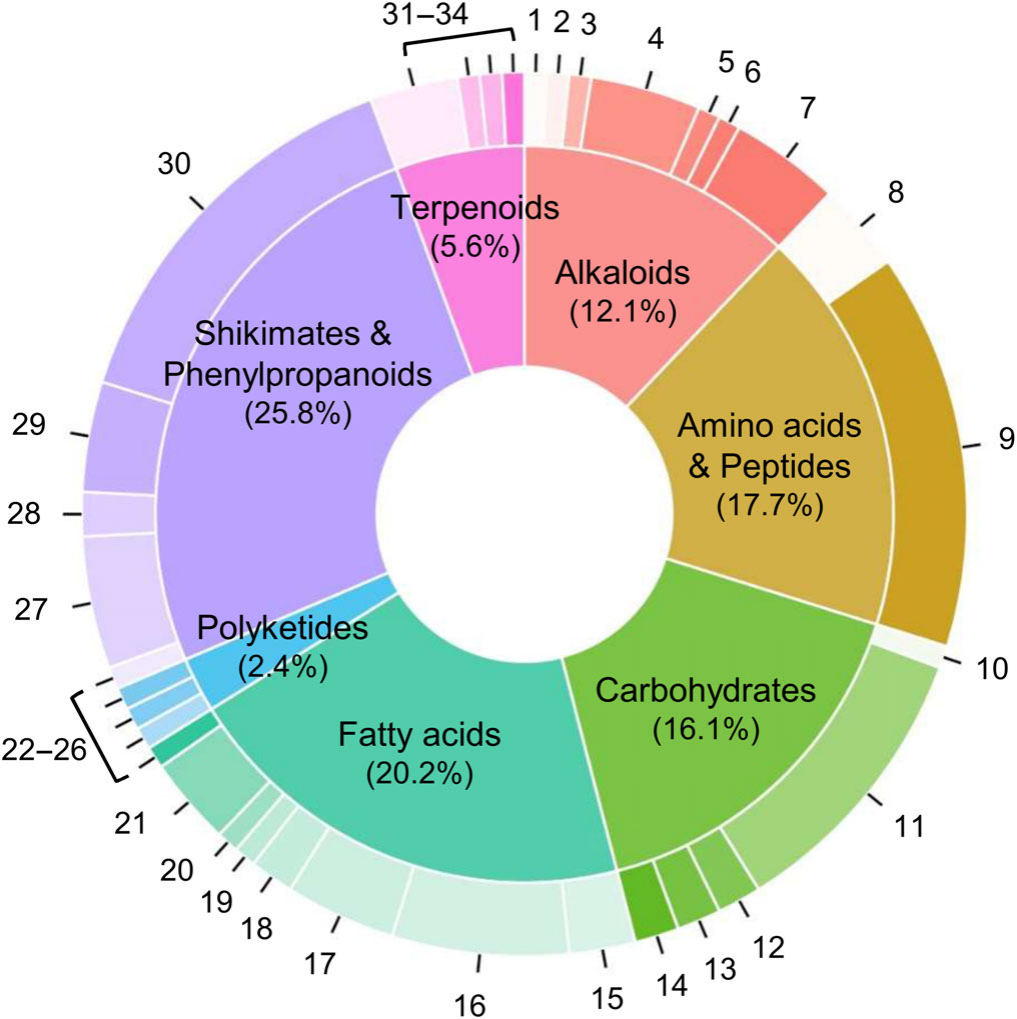
Donut chart displaying the distribution of putatively identified compounds in root exudates, categorized by the Natural Product Classification (NPC) pathway by superclass, in 105 Iberian *Arabidopsis thaliana* accessions. The size of each inner segment corresponds to the number and percentage of compounds belonging to a specific pathway, whereas the size of each outer section represents the relative abundance of compounds within each specific superclass. Compounds (following the nomenclature provided by SIRIUS 5): (1) Amino acid glycosides; (2) Anthranilic acid alkaloids; (3) Nicotinic acid alkaloids; (4) Pseudoalkaloids; (5) Serine alkaloids; (6) Small peptides; (7) Tryptophan alkaloids; (8) Amino acid glycosides; (9) Small peptides; (10) Aminosugars/Aminoglycosides; (11) Nucleosides; (12) Polyols; (13) Saccharides; (14) Small peptides; (15) Fatty acids/Conjugates; (16) Fatty acyl glycosides; (17) Fatty acyls; (18) Fatty amides; (19) Fatty esters; (20) Monoterpenoids; (21) Octadecanoids; (22) Small peptides; (23) Macrolides; (24) Phenylpropanoids; (25) Polyethers; (26) Coumarins; (27) Flavonoids; (28) Lignans; (29) Phenolic acids; (30) Phenylpropanoids; (31) Apocarotenoids; (32) Lysine alkaloids; (33) Monoterpenoids; and (34) Sesquiterpenoids.

We pinpointed 373 putative chemical compounds in root exudates from 105 Iberian *A. thaliana* accessions. In the negative ionization mode, we identified 327 compounds (range of molecular masses = 121.02–927.37 Da). In the positive mode, we identified 46 compounds (range of molecular masses = 130.05–530.98 Da). We determined the putative chemical identity and classification of 358 of these compounds with SIRIUS 5, with 124 (*c*. 33%) having a Tanimoto similarity > 50%. The NPC categorizations revealed the presence of primary metabolites, namely amino acids and peptides, carbohydrates, and fatty acids. Additionally, we detected secondary metabolite classes, including alkaloids, terpenoids, shikimates and phenylpropanoids, and polyketides. Overall, primary and secondary metabolites made up *c*. 55% and 45% of the identified compounds, respectively (Fig. 2).

We estimated the broad‐sense heritability estimates (*H* ^2^) using relative abundance data for the 373 compounds across the 105 *A. thaliana* accessions. We identified 25 compounds with *H* ^2^ values significantly different from zero, ranging between 0.26 (95% CI = 0.0421–0.305) and 0.55 (95% CI = 0.310–0.664). Up to 24 of these compounds (range of molecular masses = 145.05–577.15 Da) were chemically categorized. These compounds were primary metabolites, such as amino acids and peptides (9) and carbohydrates (8), and secondary metabolites, such as shikimates and phenylpropanoids (3), alkaloids (2), and terpenoids (2).

**Fig. 3:**
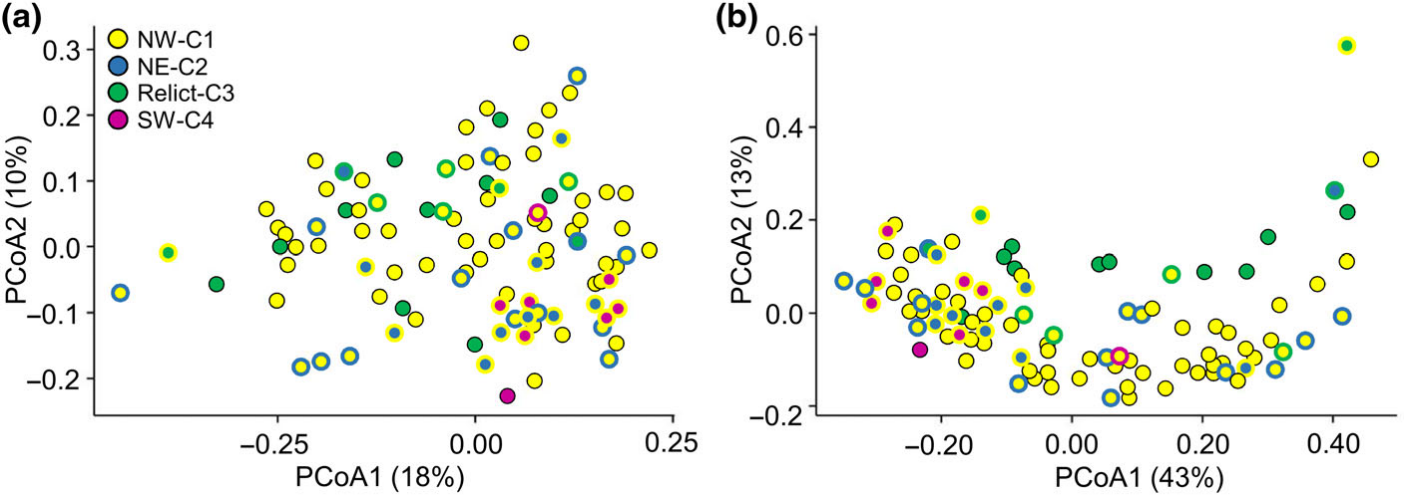
Principal coordinate analysis (PCoA) grouping 105 Iberian *Arabidopsis thaliana* accessions based on root exudates. (a) Distribution of accessions based on 373 chemical compounds. (b) Distribution of accessions based on the 25 chemical compounds with broad‐sense heritability values significantly different from zero. Accessions are color‐coded based on Fig. 1. The percentage of variation explained by the first two PCoA axes is given.

The ordination analysis for the set of 373 compounds showed that the first two PCoA axes accounted for 28% of the variance in exudate chemical composition, where some separation between clusters was observed, particularly between Relict‐C3 and SW‐C4 (Fig. 3a). This distinction held for the second ordination analysis based on the 25 compounds with *H* ^2^ values significantly different from zero (Fig. 3b). In this case, the first two PCoA axes accounted for 56% of the variance in exudate composition (Fig. 3b).

### Exploring eco‐evolutionary associations with root exudate chemical variation

We found no significant correlations between chemical distances based on the 373 putative compounds and the 25‐compound subset with *H* ^2^ values significantly different from zero, and any genetic (whole‐genome distance), geographic, environmental, or life‐history distances (*P* > 0.624 in all cases). We found the same result when compounds were categorized by their putative NPC pathways (*P* > 0.100 in all cases), except for a significant positive correlation between genetic distance and chemical distance for terpenoids (*r* = 0.225, *P* = 0.004).

The same set of analyses, but based on genetic distances using cluster proportional memberships, indicated that genetic clusters NE‐C2 and SW‐C4 did not show any significant relationships between genetic distance and chemical distance for either all compounds, or the 25‐compound subset, or NPC category combination (*P* > 0.072 in all cases). By contrast, genetic cluster Relict‐C3 did show a positively significant correlation between genetic distance and chemical distance for the 25‐compound subset (*r* = 0.100, *P* = 0.032), meaning that accessions more similar genetically were similar chemically for this set of compounds. Interestingly, genetic clusters NW‐C1 and Relict‐C3 also showed positively significant correlations between genetic distance and chemical distance for terpenoids (*r* = 0.189 and 0.216, *P* = 0.004 in both cases, for NW‐C1 and Relict‐C3, respectively).

**Fig. 4:**
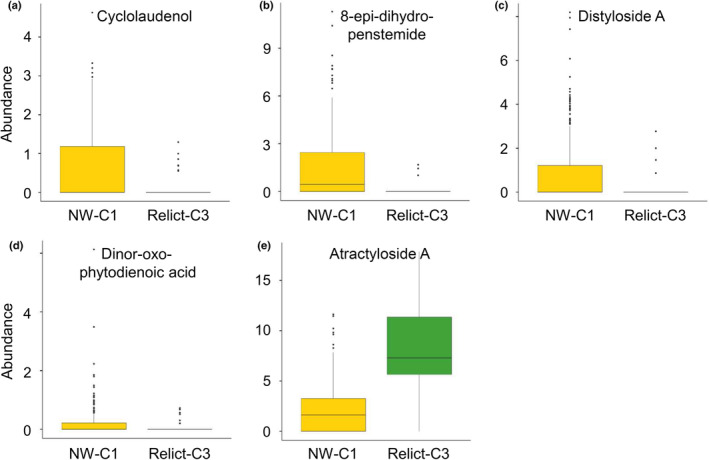
Variation in the abundance of five terpenoids (represented in a–e) found to be significant between genetic clusters NW‐C1 and Relict‐C3 in 105 Iberian *Arabidopsis thaliana* accessions. Boxes show the lower and upper quartiles, whiskers are drawn down to the 10^th^ percentile and up to the 90^th^, the line is the median of observations, and dots indicate data points falling out of percentiles. To obtain real values, numbers of axes need to be multiplied by 10^−3^ in all panels, except in panel d that need to be multiplied by 10^−2^.

We further evaluated terpenoid chemical differences between genetic clusters NW‐C1 and Relict‐C3 to detect those terpenoids further associated with their cluster proportional membership. To this end, we conducted one‐way ANOVA testing the fixed effect of genetic cluster on abundance of each of the nine terpenoids detected using 1000 runs with down‐sampled replicates (*n* = 13) to avoid bias due to sample size differences between genetic clusters (*n* = 13 and 75 for Relict‐C3 and NW‐C1, respectively). Up to five terpenoids exhibited significant differences between these two genetic clusters (*P* < 0.035 in all cases; Fig. 4). In particular, compounds distyloside A, 8‐epi‐dihydro‐penstemide, cyclolaudenol, and dinor‐oxo‐phytodienoic acid were present in NW‐C1 and practically absent in Relict‐C3, whereas atractyloside A was more abundant in Relict‐C3 than in NW‐C1 (Fig. 4).

### Genomic regions underlying chemical variation in root exudates

The GWA analyses identified 93 SNPs located in or nearby 70 genes that were significantly associated with 15 of the 109 compounds characterized in root exudates and eventually used in this analysis. Additionally, from the subset of 25 compounds with *H* ^2^ values significantly different from zero, we detected 37 SNPs linked to 10 genes. Further refinement of candidate genes based on biological pathway knowledge and GO, led to the selection of 26 genes with known functional roles aligning with 12 compounds in our dataset, including 22 genes associated with nine compounds from the larger 109‐compound dataset, and four genes linked to three compounds of the 25‐compound dataset (Table 1). These genes were classified into four broad functional categories (i.e. metabolism, signaling and regulatory networks, nutrient transport, and stress response), although some genes fell into more than one category (Table 1).

**Table 1:** Genomic associations with root exudate chemical variation in Iberian *Arabidopsis thaliana* accessions.

Family	Compound	Chr.	Position	Locus	Protein	Classification
Fatty acids^1^	N.V1092	1	1497156	AT1G05170	B3GALT2	Metabolism
Fatty acids^1^	N.V1092	1	1497156	AT1G05180	AXR1	Signaling
Carbohydrates^1^	N.V919	1	10227720	AT1G29265	MIR399A	Nutrient
Fatty acids^1^	N.V195*	1	11473826	AT1G31950	TPS29	Metab/Stress
Carbohydrates^1^	N.V919	1	12264707	AT1G33800	GXM3	Metabolism
Fatty acids^1^	N.V1092	1	17345313	AT1G47317	–	Stress
Alkaloids^2^	N.V831	1	21976138	AT1G59740	NPF4.3	Nutrient
Alkaloids^2^	N.V831	1	21976138	AT1G59740	ARF1	Signaling
Amino acids^1^	N.V313*	1	22280140	AT1G60470	GOLS4	Stress
Carbohydrates^1^	N.V554	1	22319649	AT1G60600	ABC4	Nutrient
Amino acid^2^	N.V672	2	12605976	AT2G29360	SDR	Metabolism
Amino acids^1^	N.V313	2	18065356	AT2G43500	NLP8	Nutrient
Amino acids^1^	N.V665*	3	8721212	AT3G24140	FAMA	Signaling
Amino acids^1^	N.V665*	3	8764452	AT3G24220	NCED6	Metabolism
Amino acids^1^	N.V665*	3	8769918	AT3G24225	CLE19	Signaling
Amino acids^1^	N.V665*	3	8778298	AT3G24240	RCH2	Stress
Amino acids^1^	N.V665*	3	8802982	AT3G24290	AMT1‐5	Nutrient
Amino acids^1^	N.V665*	3	8808763	AT3G24300	AMT1‐3	Nutrient
Fatty acids^1^	N.V1092	4	2395964	AT4G04720	CPK21	Stress
Shikimates^1^	N.V1001*	4	8252678	AT4G14340	CK1	Signaling
Shikimates^1^	N.V371	5	6349177	AT5G19010	MPK16	Signaling
Shikimates^1^	N.V371	5	7697150	AT5G23000	RAX1	Signaling
Shikimates^1^	N.V371	5	7714057	AT5G23010	MAM1	Metab/Stress
Shikimates^1^	N.V371	5	7719834	AT5G23020	MAM3	Metab/Stress
Carbohydrates^2^	N.V688	5	8389956	AT5G24540	BGLU31	Metab/Stress
Amino acids^1^	N.V736	5	18367078	AT5G45340	CYP707A3	Metabolism

The chemical family classification by SIRIUS 5, the specific chemical compound associated with each gene, the chromosome harboring significant SNPs, the position of the SNP in the chromosome (repeated positions indicate that SNPs were intergenic), the closest locus to the identified SNP, the protein encoded by the closest locus, and the functional classification describing the protein biological role are given. Functional classes include metabolism, signaling and regulatory networks, nutrient transport, and stress response. Superindexes 1 and 2 in the family column indicate the use of the 109‐ and 25‐compound datasets, respectively, in GWA analyses. Asterisks next to compound codes indicate that Tanimoto scores for these compounds were > 50%. Data were sorted by chromosome number and position in the chromosome. Unknown proteins indicated by dashes.

The metabolism‐related genes included those involved in the synthesis of key cell wall polysaccharides, in particular, *B3GALT2* (putative beta‐1,3‐galactosyltransferase 2) and *GXM3* (glucuronoxylan 4‐O‐methyltransferase 3). Genes related to abscisic acid (ABA) synthesis included *CYP707A3* (abscisic acid 8′‐hydroxylase 3) and *NCED6* (9‐*cis*‐epoxycarotenoid dioxygenase linked to putative t‐butoxycarbonylleucylproline). Other genes involved in secondary metabolism were those related to terpenoid (*TPS29*; terpenoid cyclases linked to putative [10E,12E]‐9,14‐dioxooctadeca‐10,12‐dienoic acid), glucosinolate biosynthesis (*MAM1*, *MAM3*; methylthioalkylmalate synthase 1 and 3, respectively), alkaloid modification (*SDR*; tropinone reductase homolog), and phenolic compound modulation (*BGLU31*; beta‐glucosidase 31).

The genes identified within the signaling and regulatory network category included transcription factors *FAMA*, *AUXIN RESPONSE FACTOR1*, *REGULATOR OF AXILLARY MERISTEMS1*, *AUXIN RESISTANT1*, a CLAVATA3/CLE‐related protein 19 (*CLE19*), casein kinase 1 (*CK1*), and mitogen‐activated protein kinase 16 (*MPK16*). These genes are integral to various biological processes, such as cell fate determination, axillary meristem regulation, auxin signaling, cell‐to‐cell communication, cell cycle control, and mitogen‐activated protein kinase signaling pathways. In this category, *FAMA* and *CLE19* were linked to putative glucobassicin, while *CK1* was linked to putative isorhamnetin 3‐glucoside‐7‐rhamnoside.

Nutrient transport‐related genes included *NPF4.3* (NRT1/PTR), a critical transporter for plant hormones; *MIR399A*, a microRNA essential for phosphate homeostasis; NIN‐like protein 8 (*NLP8*), involved in nitrogen metabolism signaling; *ABERRANT CHLOROPLAST DEVELOPMENT 4*, key in electron transport as a polyprenyltransferase; and *AMT1‐3* and *AMT1‐5*, which are important ammonium transporters. In this category, *NLP8* was linked with t‐butoxycarbonylleucylproline, whereas *AMT1‐3* and *AMT1‐5* were linked to glucobrassicin.

In the stress response category, we detected genes, such as *GOLS4*, a galactinol synthase 4, *CPK21*, a calcium‐dependent protein kinase 21, and *RGFR1*, a receptor‐like protein kinase 2. These genes play significant roles in osmoprotection, innate immunity, and calcium signaling. Notably, *BGLU31*, *MAM1*, *MAM3*, and *TPS29* can also be grouped under this category for their secondary metabolite‐induced defense functions. Here, *GOLS4* was linked with t‐butoxycarbonylleucylproline, while *RGFR1* was linked to glucobassicin.

## Discussion

Root exudates play several ecological roles that define a broad spectrum of plant responses to changes in the abiotic and biotic environment with which they interact. Notwithstanding their ecological and evolutionary value, the analysis of the amount and diversity of root exudates has been traditionally challenging for plant biologists for long‐lasting technical reasons. Because of such limitations, our understanding of the genetic and environmental factors accounting for patterns of variation in root exudates at large geographical scales, which depicts major evolutionary trajectories in heterogeneous environments, has far remained unexplored. In this study, we bridged that gap of knowledge by providing the first regional‐scale study of root exudate variation in the annual *A. thaliana* across the Iberian Peninsula, which enabled us to identify possible adaptive strategies related to biotic interactions that seem to be strongly conditioned by the genetic structure of accessions.

### Interpreting chemical complexity of *A. thaliana* root exudates

Our study identified large natural variation in chemical diversity of root exudates of *A. thaliana* across the Iberian Peninsula, including a wide range of primary (*c*. 54%) and secondary metabolites (*c*. 46%). Several primary compounds detected here were previously reported in *A. thaliana* root exudates, which may be especially critical to establish biotic interactions. For instance, malic acid is known to be involved in nutrient uptake and root–microbe interactions (Rudrappa *et al*., 2008; Ma *et al*., 2022), galactose and glucose affect rhizobacterial communities (Little *et al*., 2019; Lopes *et al*., 2022), whereas inositol plays a role in signal transduction and stress responses (Shears *et al*., 2012; Jia *et al*., 2019). Among the various amino acids and peptides detected, it is worth noting that these primary compounds in root exudates fluctuate with environmental and developmental stages (Chaparro *et al*., 2013; Strehmel *et al*., 2014). This is probably because they act as endogenous signals – influencing growth, development, primary metabolism, stress responses, and pathogen communication (Tavormina *et al*., 2015; Miller *et al*., 2017) – as well as long‐distance signaling agents (Stegmann *et al*., 2017; Takahashi & Shinozaki, 2019).

As far as secondary metabolites are concerned, they may reflect the plant's adaptive interactions with its environment, such as plant defenses and response in stress situations, as previously reported in *A. thaliana*. For instance, sinapate derivatives function as UV protectants and antimicrobial agents (Engels *et al*., 2012; Magdziak *et al*., 2020), glucopyranosides have allelopathic effects structuring the microbial community (Kimura *et al*., 2015; Upadhyay *et al*., 2022), and glucobrassicin and derivatives are well known for plant defense against herbivores and pathogens (Madloo *et al*., 2019; Soengas *et al*., 2023). In addition, sinigrin is involved in water transport under salt stress, increasing hydraulic conductance, and water permeability (Martínez‐Ballesta *et al*., 2013). It must be emphasized, however, that our untargeted approach led to putative annotations with only one‐third of the identified compounds having a Tanimoto confidence score above 0.5, which represents an important level of uncertainty in compound identification. While the methodology conducted in this study provided the first insights into root exudate composition in *A. thaliana* at a regional scale, confirming and quantifying these metabolites require targeted analyses using authentic standards (Subrahmaniam *et al*., 2023).

### Genetic background of chemical variation in root exudate composition

This study underscored the notorious natural variation in root exudate chemistry of Iberian *A. thaliana*. It is worth emphasizing that, due to technical limitations, previous studies explored genotypic variation in *A. thaliana* root exudate composition in rather limited sets of accessions (nine world‐wide accessions; Houshyani *et al*., 2012) and other genetic materials (19 MAGIC parental lines; Mönchgesang *et al*., 2016). Here, we vastly increased sample size up to 105 natural accessions across the Iberian Peninsula that enabled us to identify up to 373 putative chemical compounds. Such a step forward to quantify root exudate diversity also represented an opportunity to assess the genetic background of root exudates in *A. thaliana* in two complementary ways.

First, we were able to assess broad‐sense heritability (*H* ^2^) values of every root exudate, which is evolutionary important because they provide an estimate of the degree of genetic determination, that is the proportion of phenotypic variance explained by genotypic variance. As experiments conducted in a common environment minimize the environmental component of phenotypic variation among accessions, adaptive processes are assumed to be an important source of variation in traits with *H* ^2^ values significantly different from zero. The results showed that only 25 of 373 compounds (6.7%) exhibited *H* ^2^ values significantly different from zero, primary, and secondary metabolites constituting 67 and 33% of these 25 compounds, respectively. The greater proportion of primary metabolites with *H* ^2^ values significantly different from zero aligns with the accepted idea that natural selection is stronger on primary metabolites, which are fundamental in cell biological functions, and more relaxed on secondary metabolites, which are further related to environmental interactions (Kooke & Keurentjes, 2012). Nevertheless, the low proportion of compounds with *H* ^2^ values significantly different from zero also indicates that variation in root exudates is mostly influenced by environmental cues. Given the important and multiple direct or indirect functions of root exudates in all sort of plant responses to abiotic and biotic environmental interactions, which are per se extremely variable across space and probably over timescales, the massive proportion of both primary and secondary compounds with *H* ^2^ values indistinguishable from zero clearly reflects their environmentally driven nature.

Second, the availability of whole‐genome sequences for all Iberian *A. thaliana* accessions allowed us to explore the genomic architecture underlying regional root exudate variation, that is to detect genomic loci statistically associated with regional‐scale variation in root exudates by GWA analysis. We detected significant associations between polymorphic loci and root exudate variation in 12 compounds, identifying up to 26 genes associated with a wide spectrum of plant functions, including all major categories, such as metabolism, signaling, nutrient transport, and stress response/defense (Table 1). Although traits with high heritability normally led to the increase in statistical power for detecting causal variants in GWA analysis (Khanzadeh *et al*., 2021), we only detected four of 26 genes associated with root exudates with *H* ^2^ values significantly different from zero, pinpointing the complexity of the genetic attributes of root exudates. It is worth noting, nevertheless, that our GWA analysis must be considered as exploratory because we did not have any candidate gene to confirm, as occurs when conducting GWA analysis on *A. thaliana* traits with a strong adaptive value and a well‐known genetic basis, such as flowering time (Tabas‐Madrid *et al*., 2018).

### Eco‐evolutionary drivers of natural variation in *A. thaliana* root exudates

We assessed the role of geography, environment, life history, and genetics as major drivers of variation in chemical differences in root exudates among Iberian *A. thaliana*. Except genetics, all drivers showed nonsignificant associations with root exudate variation, indicating that macro‐environmental drivers of phenotypic variation normally used to characterize *A. thaliana* accessions across broad geographical scales cannot account for variation in root exudate composition. Despite the important environmental component of phenotypic variation detected in root exudate composition, our results suggest that environmental drivers of variation might be operating at the micro‐environmental scale, which means that micro‐environmental parameters of the spots from where accessions were sampled (e.g. vegetation type, soil composition, and temperature/humidity patterns) might better characterize such drivers of variation. Clearly, further research is needed to identify the environmental drivers of root exudate variation and the precise micro‐spatial scale at which they might be operating.

Turning to the genetic drivers of root exudate variation in Iberian A. *thaliana*, we only detected a significant relationship between genetic distance, based on whole‐genome sequences, and chemical distance for terpenoids. Perhaps because terpenoids, which are a modified class of terpenes, mediate important belowground interactions between plants and other organisms, such as microbes, herbivores, and other plants (Huang & Osbourn, 2019), they emerge as the only NPC category of root exudates with a clear association with genetic similarity among *A. thaliana* accessions. The relevance of terpenoids detected in this study is in line with a recent study on root exudate compositions using 65 plant species, where overall metabolome composition lacked a phylogenetic signal, but only phenol content in root exudates showed evidence for evolutionary conservatism (Rathore *et al*., 2023). On top of that, it must be emphasized that one of the genes detected in our GWA analysis, that is *TPS29*, is key for terpenoid synthesis within the isoprenoid pathway and is highly expressed in roots (Yu *et al*., 2020). The compound associated with *TPS29* is a fatty acid, that is (10E,12E)‐9,14‐dioxooctadeca‐10,12‐dienoic acid (Tanimoto score = 0.74), and both terpenoids and fatty acids originate from acetyl‐CoA (Sasaki & Nagano, 2004), directly linking *TPS29* to fatty acid metabolism.

Further support on the importance of terpenoids in natural root exudate variation of Iberian *A. thaliana*, and their relationship with genetic attributes, was found in the significant associations between chemical distances and genetic distances using genetic cluster membership proportions. Once again, only variation in terpenoids appeared to be significantly associated with genetic distances for genetic clusters NW‐C1 and Relict‐C3, suggesting that genetic structure also matters to account for natural variation in terpenoid exudates in *A. thaliana*. Interestingly, four terpenoids in root exudates appeared to be significantly more abundant in accessions with higher proportional memberships to NW‐C1 cluster, whereas only one clearly dominated in accessions with higher proportional memberships to Relict‐C3 cluster (Fig. 4). As Iberian clusters depict different evolutionary and demographic histories (Picó *et al*., 2008; Marcer *et al*., 2016; Tabas‐Madrid *et al*., 2018), such differences in specific terpenoids probably reflect specific evolutionary trajectories associated with each cluster. In particular, most of these terpenoids are known to be associated with plant defense in various ways, such as acting as mitochondrial inhibitors against herbivores (atractyloside A; Woyda‐Ploszczyca, 2023), displaying antimicrobial and anti‐inflammatory effects (8‐epi‐dihydro‐penstemide; Zajdel *et al*., 2012), deterring herbivores and pathogens through its potential toxicity (distyloside A; Kim *et al*., 2011), and participating in defense signaling (cyclolaudenol; Ezzat *et al*., 2016).

These findings suggest that NW‐C1 and Relict‐C3 Iberian clusters might have evolved specialized chemical defenses in response to its particular ecological challenges across their broad and heterogeneous Iberian environments. We cannot interpret the implications of these findings without falling into excessive speculation about the role of the terpenoids identified in clusters NW‐C1 and Relict‐C3, or lack thereof in clusters NE‐C2 and SW‐C4. However, our results are particularly interesting for Relict‐C3. In particular, recent studies indicated that Iberian relict *A. thaliana* exhibits unique traits not found in any other accession from all over the species' range, such as trichomes on pedicels and fruits (Arteaga *et al*., 2021, 2022), that might have also evolved as defensive barriers against a wide range of herbivores (Fürstenberg‐Hägg *et al*., 2013). Hence, the convergence of specific physical (fruit trichomes) and chemical (atractyloside A) defenses in Iberian relict *A. thaliana* suggests that interactions with herbivores/pathogens might represent an important evolutionary driver, on top of the association of this cluster with a more pronounced Mediterranean climate and nonhumanized environments (The 1001 Genomes Consortium, 2016; Marcer *et al*., 2016; Toledo *et al*., 2020). Finally, the evolutionary relevance of atractyloside A (Tanimoto score = 0.65) received further support from the fact that this compound was also the one with the highest *H* ^2^ value significantly different from zero (*H* ^2^ = 0.55), stressing the high genetic determination of this compound in Iberian *A. thaliana* and therefore its putative adaptive value. Overall, this study calls for further research on the sort of biotic interactions of relict *A. thaliana* in natural environments to understand the development of such an array of defensive strategies.

### Conclusions

Our study generated the greatest description of natural variation in root exudates known to date, identifying 373 putative compounds in 105 Iberian *A. thaliana* accessions fully characterized environmentally, genetically, and phenotypically. Interestingly, our results strongly suggest an important effect of genetic structure on root exudate composition, particularly for terpenoids, which are mostly involved in plant defenses against herbivores and pathogens. Hence, our study stresses the evolutionary importance of biotic interactions mediated by root exudates in *A. thaliana*, which aligns with other studies highlighting the role of specific *A. thaliana*'s defense metabolites, such as glucosinolates, to understand adaptive variation mediated by biotic interactions at large geographical scales (Katz *et al*., 2021). Our study represents a first step toward unraveling the enormous diversity and ecological roles of root exudates, in particular for terpenoids, which emerged as the candidate compounds to look at more carefully in the future. Nevertheless, further research needs to be conducted to improve our understanding of root exudates, including quantification of root exudates at different developmental stages (e.g. vegetative vs reproductive), environments (e.g. controlled vs natural), and stress situations (e.g. hydric, thermic, and pathogenic).

## Competing interests

None declared.

## Author contributions

HJS, MG and BKE conceived the idea. HJS, FXP, TB, MG, CLS and BKE generated materials and data. HJS, TB, BKE and FXP analyzed the data. HJS wrote the first version of the manuscript and all authors contributed to it.

## Disclaimer

The New Phytologist Foundation remains neutral with regard to jurisdictional claims in maps and in any institutional affiliations.

## Supporting information


**Dataset S1** Geography, genetics, life history, and environmental attributes of *Arabidopsis thaliana* accessions from the Iberian Peninsula.
**Dataset S2** Chemical properties, structural classifications, and heritability estimates of 373 root exudate features in *Arabidopsis thaliana* accessions.
Please note: Wiley is not responsible for the content or functionality of any Supporting Information supplied by the authors. Any queries (other than missing material) should be directed to the *New Phytologist* Central Office.

## Data Availability

Data deposited in the Dryad repository: doi: 10.5061/dryad.vmcvdnd2j (Subrahmaniam *et al*., 2024). Accession and compound data are available as Datasets S1, S2. Seeds from all accessions are publicly available through the Nottingham Arabidopsis Stock Centre (NASC; http://arabidopsis.info). Raw whole‐genome sequences are available from previous publications (The 1001 Genomes Consortium, 2016; Tabas‐Madrid *et al*., 2018). Field sampling was conducted in locations where permission was not required. Based on the Royal Decree of the Spanish legislation (Real Decreto 124/2017, de 24 de febrero; https://www.boe.es/eli/es/rd/2017/02/24/124), the genetic resources included in this study fall within the definition of ‘taxonomic purposes’.

## References

[nph20314-bib-0001] Alonso‐Blanco C , Aarts MGM , Bentsink L , Keurentjes JJB , Reymond M , Vreugdenhil D , Koornneef M . 2009. What has natural variation taught us about plant development, physiology, and adaptation? Plant Cell 21: 1877–1896. 19574434 10.1105/tpc.109.068114 PMC2729614

[nph20314-bib-0002] Arteaga N , Méndez‐Vigo B , Fuster‐Pons A , Savic M , Murillo‐Sánchez A , Picó FX , Alonso‐Blanco C . 2022. Differential environmental and genomic architectures shape the natural diversity for trichome patterning and morphology in different Arabidopsis organs. Plant, Cell & Environment 45: 3018–3035. 10.1111/pce.14308 PMC9541492 35289421

[nph20314-bib-0003] Arteaga N , Savic M , Mendez‐Vigo B , Fuster‐Pons A , Torres‐Perez R , Oliveros JC , Picó FX , Alonso‐Blanco C . 2021. MYB transcription factors drive evolutionary innovations in Arabidopsis fruit trichome patterning. Plant Cell 33: 548–565. 33955486 10.1093/plcell/koaa041 PMC8136876

[nph20314-bib-0004] Atwell S , Huang YS , Vilhjalmsson BJ , Willems G , Horton M , Li Y , Meng D , Platt A , Tarone AM , Hu TT *et al*. 2010. Genome‐wide association study of 107 phenotypes in *Arabidopsis thaliana* inbred lines. Nature 465: 627–631. 20336072 10.1038/nature08800 PMC3023908

[nph20314-bib-0005] Badri DV , de la Peña C , Lei Z , Manter DK , Chaparro JM , Guimarães RL , Sumner LW , Vivanco JM . 2012. Root secreted metabolites and proteins are involved in the early events of plant‐plant recognition prior to competition. PLoS ONE 7: e46640. 23056382 10.1371/journal.pone.0046640 PMC3462798

[nph20314-bib-0006] Badri DV , Vivanco JM . 2009. Regulation and function of root exudates. Plant, Cell & Environment 32: 666–681. 10.1111/j.1365-3040.2008.01926.x 19143988

[nph20314-bib-0007] Bates D , Mächler M , Bolker B , Walker S . 2015. Fitting linear mixed‐effects models using lme4. Journal of Statistical Software 67: 1–48.

[nph20314-bib-0008] Biedrzycki ML , Jilany TA , Dudley SA , Bais HP . 2010. Root exudates mediate kin recognition in plants. Communicative & Integrative Biology 3: 28–35. 20539778 10.4161/cib.3.1.10118 PMC2881236

[nph20314-bib-0009] Bradbury PJ , Zhang Z , Kroon DE , Casstevens TM , Ramdoss Y , Buckler ES . 2007. tassel: software for association mapping of complex traits in diverse samples. Bioinformatics 23: 2633–2635. 17586829 10.1093/bioinformatics/btm308

[nph20314-bib-0010] Brennan AC , Méndez‐Vigo B , Haddioui A , Martínez‐Zapater JM , Picó FX , Alonso‐Blanco C . 2014. The genetic structure of *Arabidopsis thaliana* in the south‐western Mediterranean range reveals a shared history between North Africa and southern Europe. BMC Plant Biology 14: 17. 24411008 10.1186/1471-2229-14-17 PMC3890648

[nph20314-bib-0011] Broadhurst D , Goodacre R , Reinke SN , Kuligowski J , Wilson ID , Lewis MR , Dunn WB . 2018. Guidelines and considerations for the use of system suitability and quality control samples in mass spectrometry assays applied in untargeted clinical metabolomic studies. Metabolomics 14: 72. 29805336 10.1007/s11306-018-1367-3 PMC5960010

[nph20314-bib-0012] Castilla AR , Méndez‐Vigo B , Marcer A , Martínez‐Minaya J , Conesa D , Picó FX , Alonso‐Blanco C . 2020. Ecological, genetic and evolutionary drivers of regional genetic differentiation in *Arabidopsis thaliana* . BMC Evolutionary Biology 20: 71. 32571210 10.1186/s12862-020-01635-2 PMC7310121

[nph20314-bib-0013] Chaparro JM , Badri DV , Bakker MG , Sugiyama A , Manter DK , Vivanco JM . 2013. Root exudation of phytochemicals in Arabidopsis follows specific patterns that are developmentally programmed and correlate with soil microbial functions. PLoS ONE 8: e55731. 23383346 10.1371/journal.pone.0055731 PMC3562227

[nph20314-bib-0014] Chen M , Yao X , Cheng H , Fan A , Lin R , Wang X , Yang Y , Chen G . 2023. Changes in Chinese fir plantations root exudation strategies seasonally and as tree age. Forest Ecology and Management 545: 121239.

[nph20314-bib-0015] van Dam NM , Bouwmeester HJ . 2017. Metabolomics in the rhizosphere: tapping into belowground chemical communication. Trends in Plant Science 21: 256–265. 10.1016/j.tplants.2016.01.008 26832948

[nph20314-bib-0016] Duan X , Zhao YY , Zhang JC . 2020. Characteristics of the root exudate release system of typical plants in plateau lakeside wetland under phosphorus stress conditions. Open Chemistry 18: 808–821.

[nph20314-bib-0017] Dührkop K , Nothias L‐F , Fleischauer M , Reher R , Ludwig M , Hoffmann MA , Petras D , Gerwick WH , Rousu J , Dorrestein PC *et al*. 2021. Systematic classification of unknown metabolites using high‐resolution fragmentation mass spectra. Nature Biotechnology 39: 462–471. 10.1038/s41587-020-0740-8 33230292

[nph20314-bib-0018] Durvasula A , Fulgione A , Gutaker RM , Alacakaptan SI , Flood PJ , Neto C , Tsuchimatsu T , Burbano HA , Picó FX , Alonso‐Blanco C *et al*. 2017. African genomes illuminate the early history and transition to selfing in *Arabidopsis thaliana* . Proceedings of the National Academy of Sciences, USA 114: 5213–5218. 10.1073/pnas.1616736114 PMC5441814 28473417

[nph20314-bib-0019] Engels C , Schieber A , Gänzle MG . 2012. Sinapic acid derivatives in defatted Oriental mustard (*Brassica juncea* L.) seed meal extracts using UHPLC‐DAD‐ESI‐MS and identification of compounds with antibacterial activity. European Food Research and Technology 234: 535–542.

[nph20314-bib-0020] Escolà Casas M , Matamoros V . 2021. Analytical challenges and solutions for performing metabolomic analysis of root exudates. Trends in Environmental Analytical Chemistry 31: e00130.

[nph20314-bib-0021] Ezzat SM , Choucry MA , Kandil ZA . 2016. Antibacterial, antioxidant, and topical anti‐inflammatory activities of *Bergia ammannioides*: a wound‐healing plant. Pharmaceutical Biology 54: 215–224. 25853974 10.3109/13880209.2015.1028079

[nph20314-bib-0022] Fang S , Clark RT , Zheng Y , Iyer‐Pascuzzi AS , Weitz JS , Kochian LV , Edelsbrunner H , Liao H , Benfey PN . 2013. Genotypic recognition and spatial responses by rice roots. Proceedings of the National Academy of Sciences, USA 110: 2670–2675. 10.1073/pnas.1222821110 PMC3574932 23362379

[nph20314-bib-0023] Fick SE , Hijmans RJ . 2017. worldclim 2: new 1‐km spatial resolution climate surfaces for global land areas. International Journal of Climatology 37: 4302–4315.

[nph20314-bib-0024] Fürstenberg‐Hägg J , Zagrobelny M , Bak S . 2013. Plant defense against insect herbivores. International Journal of Molecular Sciences 16: 10242–10297. 10.3390/ijms140510242 PMC3676838 23681010

[nph20314-bib-0025] Houshyani B , Kabouw P , Muth D , de Vos RC , Bino RJ , Bouwmeester HJ . 2012. Characterization of the natural variation in *Arabidopsis thaliana* metabolome by the analysis of metabolic distance. Metabolomics 8: 131–145. 22593725 10.1007/s11306-011-0375-3 PMC3337402

[nph20314-bib-0026] Huang AC , Osbourn A . 2019. Plant terpenes that mediate below‐ground interactions: prospects for bioengineering terpenoids for plant protection. Pest Management Science 75: 2368–2377. 30884099 10.1002/ps.5410 PMC6690754

[nph20314-bib-0027] Jia Q , Kong D , Li Q , Sun S , Song J , Zhu Y , Liang K , Ke Q , Lin W , Huang J . 2019. The function of inositol phosphatases in plant tolerance to abiotic stress. International Journal of Molecular Sciences 20: 3999. 31426386 10.3390/ijms20163999 PMC6719168

[nph20314-bib-0028] Katz E , Li JJ , Jaegle B , Ashkenazy H , Abrahams SR , Bagaza C , Holden S , Pires CJ , Angelovici R , Kliebenstein DJ . 2021. Genetic variation, environment and demography intersect to shape Arabidopsis defense metabolite variation across Europe. eLife 5: e67784. 10.7554/eLife.67784 PMC8205490 33949309

[nph20314-bib-0029] Kawasaki A , Okada S , Zhang C , Delhaize E , Mathesius U , Richardson AE , Watt M , Gilliham M , Ryan PR . 2018. A sterile hydroponic system for characterising root exudates from specific root types and whole‐root systems of large crop plants. Plant Methods 14: 114. 30598690 10.1186/s13007-018-0380-x PMC6300921

[nph20314-bib-0030] Khanzadeh H , Hossein‐Zadeh NG , Ghovvati S . 2021. Statistical power and heritability in whole‐genome association studies for quantitative traits. Meta Gene 28: 100869.

[nph20314-bib-0031] Kim HW , Wang M , Leber CA , Nothias LF , Reher R , Kang KB , van der Hooft JJJ , Dorrestein PC , Gerwick WH , Cottrell GW . 2021. NPClassifier: a deep neural network‐based structural classification tool for natural products. Journal of Natural Products 84: 2795–2807. 34662515 10.1021/acs.jnatprod.1c00399 PMC8631337

[nph20314-bib-0032] Kim JA , Yang SY , Wamiru A , McMahon JB , Le Grice SFJ , Beutler JA , Kim YH . 2011. New monoterpene glycosides and phenolic compounds from *Distylium racemosum* and their inhibitory activity against ribonuclease H. Bioorganic and Medicinal Chemistry Letters 21: 2840–2844. 21489793 10.1016/j.bmcl.2011.03.091 PMC7006235

[nph20314-bib-0033] Kimura F , Sato M , Kato‐Noguchi H . 2015. Allelopathy of pine litter: delivery of allelopathic substances into forest floor. Journal of Plant Biology 58: 61–67.

[nph20314-bib-0034] Kooke R , Keurentjes JJ . 2012. Multi‐dimensional regulation of metabolic networks shaping plant development and performance. Journal of Experimental Botany 63: 3353–3365. 22140247 10.1093/jxb/err373

[nph20314-bib-0035] Little RH , Woodcock SD , Campilongo R , Fung RKY , Heal R , Humphries L , Pacheco‐Moreno A , Paulusch S , Stigliano E , Vikeli E *et al*. 2019. Differential regulation of genes for cyclic‐di‐GMP metabolism orchestrates adaptive changes during rhizosphere colonization by *Pseudomonas* fluorescens. Frontiers in Microbiology 10: 1089. 31156596 10.3389/fmicb.2019.01089 PMC6531821

[nph20314-bib-0036] Liu W , Zhao Q , Zhang Z , Li Y , Xu N , Qu Q , Lu T , Pan X , Qian H . 2020. Enantioselective effects of imazethapyr on *Arabidopsis thaliana* root exudates and rhizosphere microbes. The Science of the Total Environment 716: 137121. 32059308 10.1016/j.scitotenv.2020.137121

[nph20314-bib-0037] Lopes LD , Wang P , Futrell SL , Schachtman DP . 2022. Sugars and jasmonic acid concentration in root exudates affect maize rhizosphere bacterial communities. Applied and Environmental Microbiology 88: e0097122. 36073926 10.1128/aem.00971-22 PMC9499034

[nph20314-bib-0038] Ludwig M , Duhrkop K , Bocker S . 2018. Bayesian networks for mass spectrometric metabolite identification via molecular fingerprints. Bioinformatics 34: i333–i340. 29949965 10.1093/bioinformatics/bty245 PMC6022630

[nph20314-bib-0039] Ludwig M , Fleischauer M , Dührkop K , Hoffmann MA , Böcker S . 2020. *De novo* molecular formula annotation and structure elucidation using SIRIUS 4. In: Li S , ed. Computational methods and data analysis for metabolomics. New York, NY, USA: Springer, 185–207. 10.1007/978-1-0716-0239-3_11 31953819

[nph20314-bib-0040] Ludwig MH , Elshamy S , Böcker S . 2012. Finding characteristic substructures for metabolite classes. German Conference on Bioinformatics 26: 23–38.

[nph20314-bib-0041] Ma W , Tang S , Dengzeng Z , Zhang D , Zhang T , Ma X . 2022. Root exudates contribute to belowground ecosystem hotspots: a review. Frontiers in Microbiology 13: 937940. 36274740 10.3389/fmicb.2022.937940 PMC9581264

[nph20314-bib-0042] Madloo P , Lema M , Francisco M , Soengas P . 2019. Role of major glucosinolates in the defense of kale against *Sclerotinia sclerotiorum* and *Xanthomonas campestris* pv. *campestris* . Phytopathology 109: 1246–1256. 30920356 10.1094/PHYTO-09-18-0340-R

[nph20314-bib-0043] Magdziak Z , Gąsecka M , Waliszewska B , Zborowska M , Mocek A , Cichy WJ , Mazela B , Kozubik T , Mocek‐Płóciniak A , Niedzielski P *et al*. 2020. The influence of environmental condition on the creation of organic compounds in *Pinus sylvestris* L. rhizosphere, roots and needles. Trees 35: 441–457.

[nph20314-bib-0044] Manzano‐Piedras E , Marcer A , Alonso‐Blanco C , Picó FX . 2014. Deciphering the adjustment between environment and life history in annuals: lessons from a geographically‐explicit approach in *Arabidopsis thaliana* . PLoS ONE 9: e87836. 24498381 10.1371/journal.pone.0087836 PMC3912251

[nph20314-bib-0045] Marcer A , Mendez‐Vigo B , Alonso‐Blanco C , Picó FX . 2016. Tackling intraspecific genetic structure in distribution models better reflects species geographical range. Ecology and Evolution 6: 2084–2097. 27066224 10.1002/ece3.2010 PMC4768750

[nph20314-bib-0046] Marcer A , Vidigal DS , James PMA , Fortin MJ , Mendez‐Vigo B , Hilhorst HWM , Bentsink L , Alonso‐Blanco C , Picó FX . 2018. Temperature fine‐tunes Mediterranean *Arabidopsis thaliana* life‐cycle phenology geographically. Plant Biology 20: 148–156. 28241389 10.1111/plb.12558

[nph20314-bib-0047] Martínez‐Ballesta MC , Muries B , Moreno DA , Dominguez‐Perles R , García‐Viguera C , Carvajal M . 2013. Involvement of a glucosinolate (sinigrin) in the regulation of water transport in *Brassica oleracea* grown under salt stress. Physiologia Plantarum 150: 145–160. 23837634 10.1111/ppl.12082

[nph20314-bib-0048] McLaughlin S , Zhalnina K , Kosina S , Northen TR , Sasse J . 2023. The core metabolome and root exudation dynamics of three phylogenetically distinct plant species. Nature Communications 14: 1649. 10.1038/s41467-023-37164-x PMC10039077 36964135

[nph20314-bib-0049] Méndez‐Vigo B , Picó FX , Ramiro M , Martínez‐Zapater JM , Alonso‐Blanco C . 2011. Altitudinal and climatic adaptation is mediated by flowering traits and *FRI*, *FLC*, and *PHYC* Genes in Arabidopsis. Plant Physiology 157: 1942–1955. 21988878 10.1104/pp.111.183426 PMC3327218

[nph20314-bib-0050] Micallef SA , Shiaris MP , Colón‐Carmona A . 2009. Influence of *Arabidopsis thaliana* accessions on rhizobacterial communities and natural variation in root exudates. Journal of Experimental Botany 60: 1729–1742. 19342429 10.1093/jxb/erp053 PMC2671628

[nph20314-bib-0051] Miller RNG , Alves GSC , Van Sluys MA . 2017. Plant immunity: unravelling the complexity of plant responses to biotic stresses. Annals of Botany 119: 681–687. 28375427 10.1093/aob/mcw284 PMC5378191

[nph20314-bib-0052] Mitchell‐Olds T , Schmitt J . 2006. Genetic mechanisms and evolutionary significance of natural variation in Arabidopsis. Nature 441: 947–952. 16791187 10.1038/nature04878

[nph20314-bib-0053] Mommer L , Kirkegaard J , van Ruijven J . 2016. Root‐root interactions: towards a rhizosphere framework. Trends in Plant Science 21: 209–217. 26832947 10.1016/j.tplants.2016.01.009

[nph20314-bib-0054] Mönchgesang S , Strehmel N , Schmidt S , Westphal L , Taruttis F , Muller E , Herklotz S , Neumann S , Scheel D . 2016. Natural variation of root exudates in *Arabidopsis thaliana*‐linking metabolomic and genomic data. Scientific Reports 6: 29033. 27363486 10.1038/srep29033 PMC4929559

[nph20314-bib-0055] Novoplansky A . 2019. What plant roots know? Seminars in Cell and Developmental Biology 92: 126–133. 30974171 10.1016/j.semcdb.2019.03.009

[nph20314-bib-0056] Oburger E , Jones DL . 2018. Sampling root exudates – mission impossible? Rhizosphere 6: 116–133.

[nph20314-bib-0057] Pantigoso HA , He Y , DiLegge MJ , Vivanco JM . 2021. Methods for root exudate collection and analysis. Methods in Molecular Biology 2232: 291–303. 33161555 10.1007/978-1-0716-1040-4_22

[nph20314-bib-0058] Picó FX , Méndez‐Vigo B , Martínez‐Zapater JM , Alonso‐Blanco C . 2008. Natural genetic variation of *Arabidopsis thaliana* is geographically structured in the Iberian Peninsula. Genetics 180: 1009–1021. 18716334 10.1534/genetics.108.089581 PMC2567352

[nph20314-bib-0059] R Core Team . 2021. R: a language and environment for statistical computing. Vienna, Austria: R Foundation for Statistical Computing.

[nph20314-bib-0060] Rasmann S , Hiltpold I . 2022. Root exudation of specialized molecules for plant‐environment interaction. Chimia 76: 922–927. 38069787 10.2533/chimia.2022.922

[nph20314-bib-0061] Rathore N , Hanzelková V , Dostálek T , Semerád J , Schnablová R , Cajthaml T , Münzbergová Z . 2023. Species phylogeny, ecology, and root traits as predictors of root exudate composition. New Phytologist 239: 1212–1224. 37421208 10.1111/nph.19060

[nph20314-bib-0062] Rosenberg MS , Anderson CD . 2011. PASSaGE: pattern analysis, spatial statistics and geographic exegesis, v.2. Methods in Ecology and Evolution 2: 229–232.

[nph20314-bib-0063] Rudrappa T , Czymmek KJ , Paré PW , Bais HP . 2008. Root‐secreted malic acid recruits beneficial soil bacteria. Plant Physiology 148: 1547–1556. 18820082 10.1104/pp.108.127613 PMC2577262

[nph20314-bib-0064] Sasaki Y , Nagano Y . 2004. Plant Acetyl‐CoA carboxylase: structure, biosynthesis, regulation, and gene manipulation for plant breeding. Bioscience, Biotechnology, and Biochemistry 68: 1175–1184. 15215578 10.1271/bbb.68.1175

[nph20314-bib-0065] Schmid R , Heuckeroth S , Korf A , Smirnov A , Myers O , Dyrlund TS , Bushuiev R , Murray KJ , Hoffmann N , Lu M *et al*. 2023. Integrative analysis of multimodal mass spectrometry data in MZmine 3. Nature Biotechnology 41: 447–449. 10.1038/s41587-023-01690-2 PMC10496610 36859716

[nph20314-bib-0066] Shears SB , Ganapathi SB , Gokhale NA , Schenk TMH , Wang H , Weaver JD , Zaremba A , Zhou Y . 2012. Defining signal transduction by inositol phosphates. In: Balla T , Wymann M , York JD , eds. Phosphoinositides II: the diverse biological functions. Dordrecht, the Netherlands: Springer, 389–412. 10.1007/978-94-007-3015-1_13 PMC3925325 22374098

[nph20314-bib-0067] Soengas P , Madloo P , Lema M . 2023. Spectral reflectance indexes reveal differences in the physiological status of *Brassica oleracea* with contrasting glucosinolate content under biotic stress. Plants 12: 2698. 37514312 10.3390/plants12142698 PMC10384497

[nph20314-bib-0068] Song F , Han X , Zhu X , Herbert SJ . 2012. Response to water stress of soil enzymes and root exudates from drought and non‐drought tolerant corn hybrids at different growth stages. Canadian Journal of Soil Science 92: 501–507.

[nph20314-bib-0069] Stegmann M , Monaghan J , Smakowska‐Luzan E , Rovenich H , Lehner A , Holton N , Belkhadir Y , Zipfel C . 2017. The receptor kinase FER is a RALF‐regulated scaffold controlling plant immune signaling. Science 355: 287–289. 28104890 10.1126/science.aal2541

[nph20314-bib-0070] Strehmel N , Böttcher C , Schmidt S , Scheel D . 2014. Profiling of secondary metabolites in root exudates of *Arabidopsis thaliana* . Phytochemistry 108: 35–46. 25457500 10.1016/j.phytochem.2014.10.003

[nph20314-bib-0071] Subrahmaniam HJ , Lind Salomonsen C , Radutoiu S , Ehlers BK , Glasius M . 2023. Unraveling the secrets of plant roots: simplified method for large scale root exudate sampling and analysis in *Arabidopsis thaliana* . Open Research Europe 3: 12. 37645513 10.12688/openreseurope.15377.3 PMC10445920

[nph20314-bib-0072] Subrahmaniam HJ , Picó FX , Bataillon T , Salomonsen CL , Glasius M , Ehlers BK . 2024. Natural variation in root exudate composition in the genetically structured *Arabidopsis thaliana* in the Iberian Peninsula. Dryad Digital Repository. doi: 10.5061/dryad.vmcvdnd2j. PMC11754937 39658885

[nph20314-bib-0074] Tabas‐Madrid D , Mendez‐Vigo B , Arteaga N , Marcer A , Pascual‐Montano A , Weigel D , Picó FX , Alonso‐Blanco C . 2018. Genome‐wide signatures of flowering adaptation to climate temperature: regional analyses in a highly diverse native range of *Arabidopsis thaliana* . Plant, Cell & Environment 41: 1806–1820. 10.1111/pce.13189 29520809

[nph20314-bib-0075] Takahashi F , Shinozaki K . 2019. Long‐distance signaling in plant stress response. Current Opinion in Plant Biology 47: 106–111. 30445314 10.1016/j.pbi.2018.10.006

[nph20314-bib-0076] Tavormina P , De Coninck B , Nikonorova N , De Smet I , Cammue BP . 2015. The Plant peptidome: an expanding repertoire of structural features and biological functions. Plant Cell 27: 2095–2118. 26276833 10.1105/tpc.15.00440 PMC4568509

[nph20314-bib-0077] The 1001 Genomes Consortium . 2016. 1135 Genomes reveal the global pattern of polymorphism in *Arabidopsis thaliana* . Cell 166: 481–491. 27293186 10.1016/j.cell.2016.05.063 PMC4949382

[nph20314-bib-0078] Toledo B , Marcer A , Méndez‐Vigo B , Alonso‐Blanco C , Picó FX . 2020. An ecological history of the relict genetic lineage of *Arabidopsis thaliana* . Environmental and Experimental Botany 170: 103800.

[nph20314-bib-0079] Upadhyay SK , Srivastava AK , Rajput VD , Chauhan PK , Bhojiya AA , Jain D , Chaubey G , Dwivedi P , Sharma B , Minkina T . 2022. Root exudates: mechanistic insight of plant growth promoting rhizobacteria for sustainable crop production. Frontiers in Microbiology 13: 916488. 35910633 10.3389/fmicb.2022.916488 PMC9329127

[nph20314-bib-0080] Vidigal DS , Marques AC , Willems LA , Buijs G , Mendez‐Vigo B , Hilhorst HW , Bentsink L , Picó FX , Alonso‐Blanco C . 2016. Altitudinal and climatic associations of seed dormancy and flowering traits evidence adaptation of annual life cycle timing in *Arabidopsis thaliana* . Plant, Cell & Environment 39: 1737–1748. 10.1111/pce.12734 26991665

[nph20314-bib-0081] Vives‐Peris V , de Ollas C , Gómez‐Cadenas A , Pérez‐Clemente RM . 2020. Root exudates: from plant to rhizosphere and beyond. Plant Cell Reports 39: 3–17. 31346716 10.1007/s00299-019-02447-5

[nph20314-bib-0082] Wang NQ , Kong CH , Wang P , Meiners SJ . 2021. Root exudate signals in plant–plant interactions. Plant, Cell & Environment 44: 1044–1058. 10.1111/pce.13892 32931018

[nph20314-bib-0083] Wang S , Su Y , Li J , Lu Y , Mei X , Wang J . 2022. Integration of LC/MS‐based molecular networking and molecular docking allows in‐depth annotation and prediction of the metabolome: a study of *Salvia miltiorrhiza* Bunge. Industrial Crops and Products 186: 115298.

[nph20314-bib-0084] Wen T , Zhao M , Yuan J , Kowalchuk GA , Shen Q . 2021. Root exudates mediate plant defense against foliar pathogens by recruiting beneficial microbes. Soil Ecology Letters 3: 42–51.

[nph20314-bib-0085] Williams A , de Vries FT . 2020. Plant root exudation under drought: implications for ecosystem functioning. New Phytologist 225: 1899–1905. 31571220 10.1111/nph.16223

[nph20314-bib-0086] Woyda‐Ploszczyca AM . 2023. Direct and indirect targets of carboxyatractyloside, including overlooked toxicity toward nucleoside diphosphate kinase (NDPK) and mitochondrial H^+^ leak. Pharmaceutical Biology 61: 372–390. 36799406 10.1080/13880209.2023.2168704 PMC9946330

[nph20314-bib-0087] Yu Z , Zhao C , Zhang G , Teixeira da Silva JA , Duan J . 2020. Genome‐wide identification and expression profile of TPS gene family in Dendrobium officinale and the role of DoTPS10 in linalool biosynthesis. International Journal of Molecular Sciences 21: 5419. 32751445 10.3390/ijms21155419 PMC7432446

[nph20314-bib-0088] Zajdel SM , Graikou K , Głowniak K , Chinou I . 2012. Chemical analysis of *Penstemon campanulatus* (Cav.) Willd. — antimicrobial activities. Fitoterapia 83: 373–376. 22155592 10.1016/j.fitote.2011.11.021

[nph20314-bib-0089] Zulfiqar M , Gadelha L , Steinbeck C , Sorokina M , Peters K . 2023. MAW: the reproducible Metabolome Annotation Workflow for untargeted tandem mass spectrometry. Journal of Cheminformatics 15: 32. 36871033 10.1186/s13321-023-00695-y PMC9985203

